# Can soy isoflavones in combination with soy protein change serum concentration of adiponectin and resistin? A systematic review and meta‐analysis on randomized clinical trials

**DOI:** 10.1002/fsn3.3038

**Published:** 2022-09-15

**Authors:** Mitra Hariri, Bahareh Amirkalali, Ensiyeh Mollanoroozy, Ali Gholami

**Affiliations:** ^1^ Noncommunicable Diseases Research Center Neyshabur University of Medical Sciences Neyshabur Iran; ^2^ Gastrointestinal & Liver Diseases Research Center Iran University of Medical Sciences Tehran Iran; ^3^ Department of Epidemiology and Biostatistics, School of Public Health Neyshabur University of Medical Sciences Neyshabur Iran

**Keywords:** adiponectin, meta‐analysis, resistin, soy isoflavones, soy protein, systematic review

## Abstract

Some studies proposed the anti‐inflammatory effect of soy protein and soy isoflavones by changing the serum adiponectin and resistin levels. The purpose of this research was to determine the impact of soy isoflavones and soy protein on blood adiponectin and resistin levels in adults. Scopus, PubMed, Cochrane Library, ISI Web of Science, and ClinicalTrials.gov databases were searched until April 2022. The effect size was computed by the mean changes from the beginning for intervention and comparison groups and their standard deviation. In the case of significant heterogeneity, DerSimonian and Laird random‐effects model was used. Six and five clinical trials were selected for the systematic review and meta‐analysis, respectively. The overall estimate indicated that soy isoflavones in combination with soy protein did not significantly change serum adiponectin level (weighted mean differences (WMD) = 0.36 μg/ml; 95% confidence interval (CI): −0.26, 0.99; *p* = .25), but significantly increased serum resistin level (WMD = 0.64 ng/ml, 95% CI: 0.25, 1.04; *p* = .001). In combination with soy protein, soy isoflavones nonsignificantly increased serum adiponectin levels, but significantly increased resistin levels. New intervention studies with a high dose of soy isoflavones and soy protein in different parts of the world and an updated meta‐analysis are needed to confirm the results of our study.

## Graphical abstract

Soy isoflavones in combination with soy protein non‐significantly increased serum adiponectin levels, but significantly increased resistin levels.

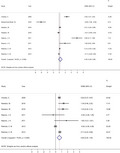

## 1 INTRODUCTION

White adipose tissue is considered a metabolically active and endocrine organ. It is the source of various bioactive and endocrine compounds known as adipokines (Coelho et al., 2013). Adipokines are involved in many physiological and pathological processes which finally determine individuals' health conditions (Berg et al., 2001; Comninos et al., 2014; Rak et al., 2017; Yamauchi et al., 2001).

Adiponectin is the most abundant circulatory adipokine and has a crucial role in suppressing the metabolic disorders which may result in non‐alcoholic fatty liver disease (NAFLD), obesity, metabolic syndrome, type 2 diabetes mellitus (T2DM), and atherosclerosis (Abou‐Samra et al., 2020; Achari & Jain, 2017; Dıez & Iglesias, 2003; Renaldi et al., 2009; Stern et al., 2016). It does so primarily by reducing hepatic glucose synthesis and regulating fatty acid oxidation (Fisman & Tenenbaum, 2014).

Considerable studies have reported a positive correlation between resistin and insulin (Jung & Choi, 2014; Zhang et al., 2008). Moreover, downregulation of resistin at the genetic level improves insulin sensitivity and glucose metabolism (Lazar, 2007). Its circulatory level was positively correlated with the levels of inflammatory biomarkers, such as tumor necrosis factor‐α (TNF‐α), c‐reactive protein (CRP), and interleukin‐6 (IL‐6), and some chronic diseases, such as chronic kidney disease, rheumatoid arthritis, and coronary atherosclerosis (Park & Ahima, 2013).

Several studies indicated that various functional foods or dietary components (such as soybean) can affect the function or expression of these adipokines (Flachs et al., 2006; Mellouk et al., 2018; Nagasawa et al., 2002; Zhang et al., 2008). Soybean belongs to the legume family that is made up of protein (36–40%, mostly glycinin and β‐conglycinin), lipids (20%), carbohydrates (30–35%), and some important minor components such as phytic acid and isoflavones (the major ones: aglycones, genistein, daidzein, and glycitein) (Garcia et al., 1997; Ko, 2014).

Scientists indicated that soy isoflavones and protein play an anti‐inflammatory role by increasing adiponectin (Charles et al., 2009). However, other scientists did not indicate this effect (Christie et al., 2010; Mohammad‐Shahi et al., 2016; Nadadur et al., 2016; Napora et al., 2011; Rebholz et al., 2013).

Recently, a systematic review and meta‐analysis has suggested that soy products have no significant effect on serum adiponectin levels in the adults (Moosavian et al., 2021). Regarding diverse amounts of isoflavones and proteins in different soy products, this study is considered to be not capable of investigating the effects of these two components on serum concentration of adiponectin.

There are limited randomized clinical trials (RCTs) on the effect of soy protein and soy isoflavones on resistin level, which produce contradictory results (Charles et al., 2009; Nadadur et al., 2016; Napora et al., 2011; Rebholz et al., 2013), whereas no systematic review and meta‐analysis have reported the effect of soy isoflavones containing soy protein on resistin levels.

Due to inconsistent findings regarding the effect of soy isoflavones and soy protein on serum adiponectin and resistin levels and the absence of a comprehensive systematic review and meta‐analysis on this subject, this study will conduct a systematic review of clinical trials and a meta‐analysis to investigate the effect of soy isoflavones and soy protein on serum adiponectin and resistin levels.

## 2 MATERIALS AND METHODS

Preferred Reporting Items for Systematic Reviews and Meta‐Analyses (PRISMA) guidelines were used for all stages of design, implementation, and reporting in this study (Moher et al., 2009). Study protocol was registered in PROSPERO (No. CRD42021228366).

### 2.1 Literature search

To find the RCTs assessing soy isoflavones in combination with soy protein effect on serum adiponectin and resistin levels, Scopus, PubMed, Cochrane Library, ISI Web of Science, and ClinicalTrials.gov were searched until April 2022. The RCTs were examined with no limitation on publication date. Our search was designed by following terms: “Soy Food,” “Isoflavones,” “Soybean Proteins,” “Adipokines,” “Adiponectin,” “Resistin,” and “Clinical Trials.” If the databases were searched based on the keywords of adiponectin and resistin, few articles would be missed. To this end, the most relevant and important adipokines keywords were employed. Search stretchy was designed by Boolean operators (AND and OR). We also used parentheses, quotation marks, and asterisks for searching group search terms, the exact word, and all words made of one word, respectively (Table S1).

All found papers were imported to EndNote (reference manager software, version X9) after removing duplicate papers, the titles and abstracts were read by two independent reviewers (AGh, MH). Moreover, we reviewed the reference lists of all relevant reviews on this issue and all included RCTs to round up our search. We contacted the appropriate authors to get clarity on any confusing material. A group discussion was used to settle disagreements.

### 2.2 Inclusion criteria

Inclusion criteria were outlined in terms of PICOS framework: (1) Population: Participants aged more than 18 years old with no gender‐based and health restriction; (2) Intervention: the combination of soy isoflavones and soy protein; (3) Comparator: Comparison group; (4) Outcomes: Measuring adiponectin and resistin; (5) Study design: RCTs.

### 2.3 Exclusion criteria

Papers were excluded from this study as follows: (1) unclear information for serum concentration of adiponectin and resistin; (2) measuring postprandial levels of adiponectin or resistin; (3) not reporting the intervention duration; (4) taking food supplements besides soy isoflavones plus soy proteins; (5) not having a comparison group; (6) not having data for the dose of soy isoflavones and soy protein, and (7) non‐English articles.

### 2.4 Data extraction

After finalizing RCTs eligibility to be included, the following information was extracted by two independent reviewers (AGh, MH): first author's last name, soy isoflavones dose, study design, soy protein dose, study duration, health status, number of subjects in intervention and comparison groups, the country where RCTs was conducted, participants' sex, age, body mass index (BMI), RCT publication year, mean and standard deviation (SD) of serum concentration of adiponectin and resistin. We converted all units for adiponectin and resistin into the same unit (μg/ml for adiponectin and ng/ml for resistin). RCTs with more than one group as a comparison group and more than one group as an intervention group were divided into two or more RCTs. If any discrepancy was found on extracted information, researchers consulted again and MH sent an email to corresponding authors to clarify unclear information.

### 2.5 Quality assessment

According to the items of Cochrane Collaboration's tool, the quality of eligible RCTs was assessed by two independent reviewers (MH, BA) ( Higgins & Green, 2009): (I) blinding of participants and personnel; (II) blinding of outcome assessment; (III) allocation concealment; (IV) random sequence generation; (V) incomplete reporting of results; and (VI) selective reporting. The risk of bias for each item was judged as “high,” “low,” and “unclear.” RCTs which had at least three, two, and less than two items with low risk of bias were scored “good” and “fair,” and “weak,” respectively.

### 2.6 Statistical analysis

A meta‐analysis was performed by calculating mean differences (MDs) and their SDs for adiponectin and resistin through extracting data from included articles.

According to Cochrane Handbook, effect sizes were calculated via mean changes from the beginning for adiponectin and resistin levels and their SDs for both study groups (Higgins & Green, 2009). Since median or range might be reported instead of the mean of adiponectin and resistin concentrations, Hozo method (Hozo et al., 2005) was used to estimate the mean. If studies reported standard errors (SEs), SDs were calculated by multiplying SEs by the square root of the study sample size. In the case of significant heterogeneity of the intervention effects, DerSimonian and Laird random‐effects model was applied to estimate the summary of the overall effects (DerSimonian & Laird, 1986). We calculated the statistical heterogeneity via Cochran's Q test and I‐squared statistic. A *p*‐value ≤ .10 and value ≥50% were considered as significant heterogeneity for the Cochran's Q test and the *I*‐squared statistic, respectively (Higgins & Thompson, 2002). Subgroup analyses were conducted based on study design, mean baseline serum concentrations of adiponectin and resistin, dose of soy isoflavones and soy protein, article quality assessment, intervention duration, health status, participants' sex, age, BMI, study publication year, sample size, and geographical region to determine the source of heterogeneity. The presence of publication bias in the meta‐analysis was checked by Eggar's weighted regression tests, Begg's funnel plot, and Begg's rank correlation (Begg & Berlin, 1989; Egger et al., 1997). Sensitivity analysis was used to evaluate the effect of each study on the overall effect. We used STATA 15 (Stata Corp, College Station, TX, USA) to conduct statistical analyses. The statistical significance was considered at 0.05, and all calculated effect sizes were presented in 95% confidence intervals (CI).

## 3 RESULTS

### 3.1 Study selection

**FIGURE 1:**
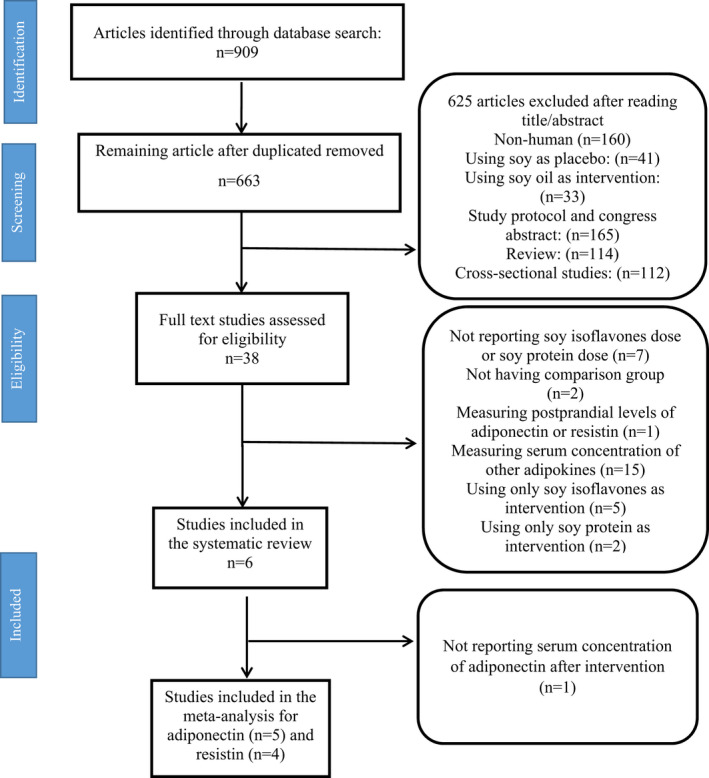
Flowchart of study selection process

After a systematic search of five databases, 909 papers were retrieved from which 246 papers were duplicated. As a result of reviewing the titles and abstracts of 663 papers, 625 were excluded from the study for a variety of reasons, including the use of soy oil as an intervention, being congress abstracts, being non‐human studies, using soy as a placebo, study protocols, reviews, and cross‐sectional studies. We read the full text of 38 articles and excluded 32 articles from our study for the following reasons: without any information related to the dose of soy protein or soy isoflavones (*n* = 7), without any comparison group (*n* = 2), measuring postprandial levels of adiponectin or resistin (*n* = 1), measuring the serum concentration of other adipokines (*n* = 15), using only soy isoflavones as intervention (*n* = 5), and using only soy protein as intervention (*n* = 2). Finally, 6 RCTs were selected for the systematic reviews (Charles et al., 2009; Christie et al., 2010; Mohammad‐Shahi et al., 2016; Nadadur et al., 2016; Napora et al., 2011; Rebholz et al., 2013). Since one RCT reported no information for serum concentration of adiponectin after the intervention, it was not included in this meta‐analysis (Christie et al., 2010); therefore, five articles were remained to be included in adiponectin meta‐analysis (Charles et al., 2009; Mohammad‐Shahi et al., 2016; Nadadur et al., 2016; Napora et al., 2011; Rebholz et al., 2013) and four articles were selected for resistin meta‐analysis (Charles et al., 2009; Nadadur et al., 2016; Napora et al., 2011; Rebholz et al., 2013) (Figure 1).

### 3.2 Study characteristics

Six articles studied the effect of soy isoflavones combined with soy protein on serum adiponectin (Charles et al., 2009; Christie et al., 2010; Mohammad‐Shahi et al., 2016; Nadadur et al., 2016; Napora et al., 2011; Rebholz et al., 2013), and four studies assessed the effect of soy isoflavones in combination with soy protein on serum resistin (Charles et al., 2009; Nadadur et al., 2016; Napora et al., 2011; Rebholz et al., 2013).

Regarding the number of groups and assessment periods, two studies had two comparison groups (Nadadur et al., 2016; Rebholz et al., 2013) and one study reported the concentration of serum adiponectin in two intervals during intervention period (Napora et al., 2011), thus, each one of these articles was considered as two independent articles with separate effect sizes.

Concerning the study design, two and four studies had cross‐over (Mohammad‐Shahi et al., 2016; Rebholz et al., 2013) and parallel designs, respectively (Charles et al., 2009; Christie et al., 2010; Nadadur et al., 2016; Napora et al., 2011). Treatment duration was ranged from 4 to 48 weeks and intervention doses for soy protein and soy isoflavones were 7–40 g/day and 17–160 mg/day, respectively. Regarding health status, two studies were conducted on healthy postmenopausal women (Charles et al., 2009; Nadadur et al., 2016), one study on overweight or obese postmenopausal women (Christie et al., 2010), one study on women with rheumatoid arthritis (Mohammad‐Shahi et al., 2016), one study on men with prostate cancer (Napora et al., 2011) and one study on hypertensive individuals (Rebholz et al., 2013).

One study was not included in the current meta‐analysis among six studies because no information for serum adiponectin level after the intervention was reported (Christie et al., 2010). Therefore, five studies with a total of 15 datasets (eight datasets for the effect of soy isoflavones in combination with soy protein on serum adiponectin, and seven datasets for the effect of soy isoflavones in combination with soy protein on serum resistin) were included in this meta‐analysis. Details of the study characteristics are presented in Table 1.

**TABLE 1:** Randomized controlled trial studies included in the systematic review and meta‐analysis

Code, Author (year) (country)	Subjects	Age and BMI (mean ± SD)	RCT	Intervention	Placebo	Duration (week)	Variables	Results
1 Charles, C 2009 USA	Healthy postmenopausal women *N* = 75	Age: 57.3 ± 1.10 BMI: 26.4 ± 0.9	Randomized, double‐blind, placebo‐controlled trial	20 g of soy protein with 160 mg of total isoflavones	20 g/day whole milk protein	12	Adiponectin and Resistin	Serum concentration of adiponectin increased significantly in intervention group. There was no significant change in serum concentration of resistin between two groups
2 Christie, D. R. 2010 England	Obese postmenopausal white and African American women *N* = 33	Age: 54.4 ± 3.3 BMI: 35.3 ± 6.0	Randomized, double‐blind, controlled trial	Soy shakes (supplied 20 g soy protein plus 160 mg isoflavones)	Casein without isoflavones	12	Adiponectin	There was no significant change in serum concentration of adiponectin between two groups
3 Mohammad‐Shahi, M. 2016 Iran	Women with rheumatoid arthritis *N* = 25	Age: 45.72 ± 55.45 BMI: 29.62 ± 5.85	Randomized, crossover clinical trial	Soy milk (supplied 7 g/day soy protein plus 17 mg/day isoflavones)	Dairy milk	4	Adiponectin	There was no significant change in serum concentration of adiponectin between two groups
4.1 Nadadur, M. 2016 USA	Healthy postmenopausal women *N* = 37	Age: 56.9 ± 6.1 BMI: N/M	Single blind randomized clinical trial	Soy food diet (containing 15 g/day soy protein and 50 mg/day soy isoflavones	Very low fat diet	8	Adiponectin and Resistin	There was no significant change in serum concentration of adiponectin and resistin between two groups
4.2 Nadadur, M. 2016 USA	Healthy postmenopausal women *N* = 37	Age: 56.9 ± 6.1 BMI: N/M	Single blind randomized clinical trial	Soy food diet (containing 15 g/day soy protein and 50 mg/day soy isoflavones	Control diet	8	Adiponectin and Resistin	There was no significant change in serum concentration of adiponectin and resistin between two groups
5.1 Napora, J. K. 2011 USA	Men with prostate cancer N:33	Age: 69.2 ± 13.69 BMI: 28.71 ± 7.1	Randomized, double‐blind, placebo‐controlled	20 g of soy protein containing 160 mg of total isoflavones	20 g whole milk protein	6	Adiponectin and Resistin	There was no significant change in serum concentration of adiponectin and resistin between two groups
5.2 Napora, J. K. 2011 USA	Men with prostate cancer N:33	Age: 69.2 ± 13.69 BMI: 28.71 ± 7.1	Randomized, double‐blind, placebo‐controlled	20 g of soy protein containing 160 mg of total isoflavones	20 g whole milk protein	12	Adiponectin and Resistin	There was no significant change in serum concentration of adiponectin and resistin between two groups
6.1 Rebholz, C. M 2013 USA	Hypertensive individuals *N* = 48	Age: 48.2 ± 11.7 BMI: 29.5 ± 3.8	Randomized, placebo‐controlled, double‐blind, three‐phase crossover trial	40 g of soybean protein supplement (supplied 89.3 mg/day isoflavones)	Complex carbohydrate supplements	8	Adiponectin and Resistin	There was no significant change in serum concentration of adiponectin and resistin between two groups
6.2 Rebholz, C. M 2013 USA	Hypertensive individuals *N* = 51	Age: 48.2 ± 11.7 BMI: 29.5 ± 3.8	Randomized, placebo‐controlled, double‐blind, three‐phase crossover trial	40 g of soybean protein supplement (supplied 89.3 mg/day isoflavones)	Milk protein	8	Adiponectin and Resistin	There was no significant change in serum concentration of adiponectin and resistin between two groups

*Note*: Mean (IQ), Means (95% confidence interval).

Abbreviations: BMI, Body Mass Index; N/M, Not mention; RCT, Randomized clinical trial.

### 3.3 Risk of bias assessment

Based on Cochrane guidelines, from a total of six studies, four studies were scored as “Good” (Charles et al., 2009; Christie et al., 2010; Napora et al., 2011; Rebholz et al., 2013), and two studies as “weak” (Mohammad‐Shahi et al., 2016; Nadadur et al., 2016). Allocation concealment, blinding of participants and personnel, blinding of outcome assessment, incomplete outcome, and selective reporting were the sources of high risk of bias in one (Nadadur et al., 2016), one (Mohammad‐Shahi et al., 2016), two (Charles et al., 2009; Nadadur et al., 2016), two (Charles et al., 2009; Nadadur et al., 2016), and one (Christie et al., 2010) studies. More information is presented in detail in Table 2.

**TABLE 2:** Quality of bias assessment of the included studies according to the Cochrane guidelines

Author name, year of publication, references	Random sequence generation	Allocation concealment	Blinding of participants and personnel	Blinding of outcome assessment	Incomplete outcome data	Selective reporting	Overall quality
Charles et al. (2009)	L	L	L	L	H	U	Good
Christie et al. (2010)	L	L	L	U	L	H	Good
Mohammad‐Shahi et al. (2016)	U	U	H	H	L	U	Weak
Nadadur et al. (2016)	U	H	U	H	H	U	Weak
Napora et al. (2011)	L	U	L	U	L	U	Good
Rebholz et al. (2013)	L	U	L	L	U	L	Good

Abbreviations: L, low risk of bias; H, high risk of bias; U, unclear risk of bias.

### 3.4 Findings of the meta‐analysis

**FIGURE 2:**
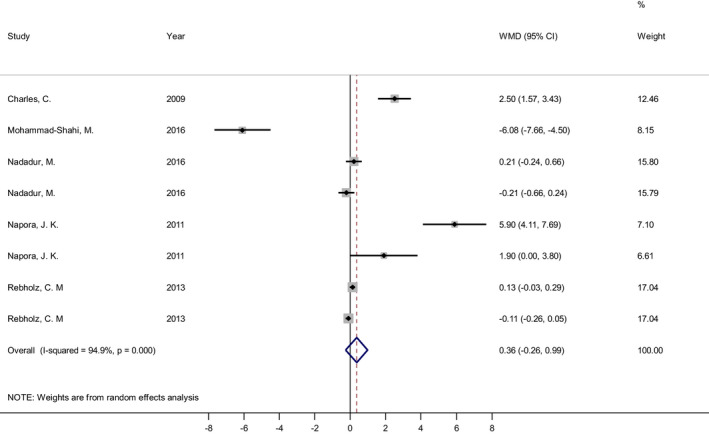
Forest plot of the effect of soy isoflavones plus soy protein consumption on serum concentrations of adiponectin

**FIGURE 3:**
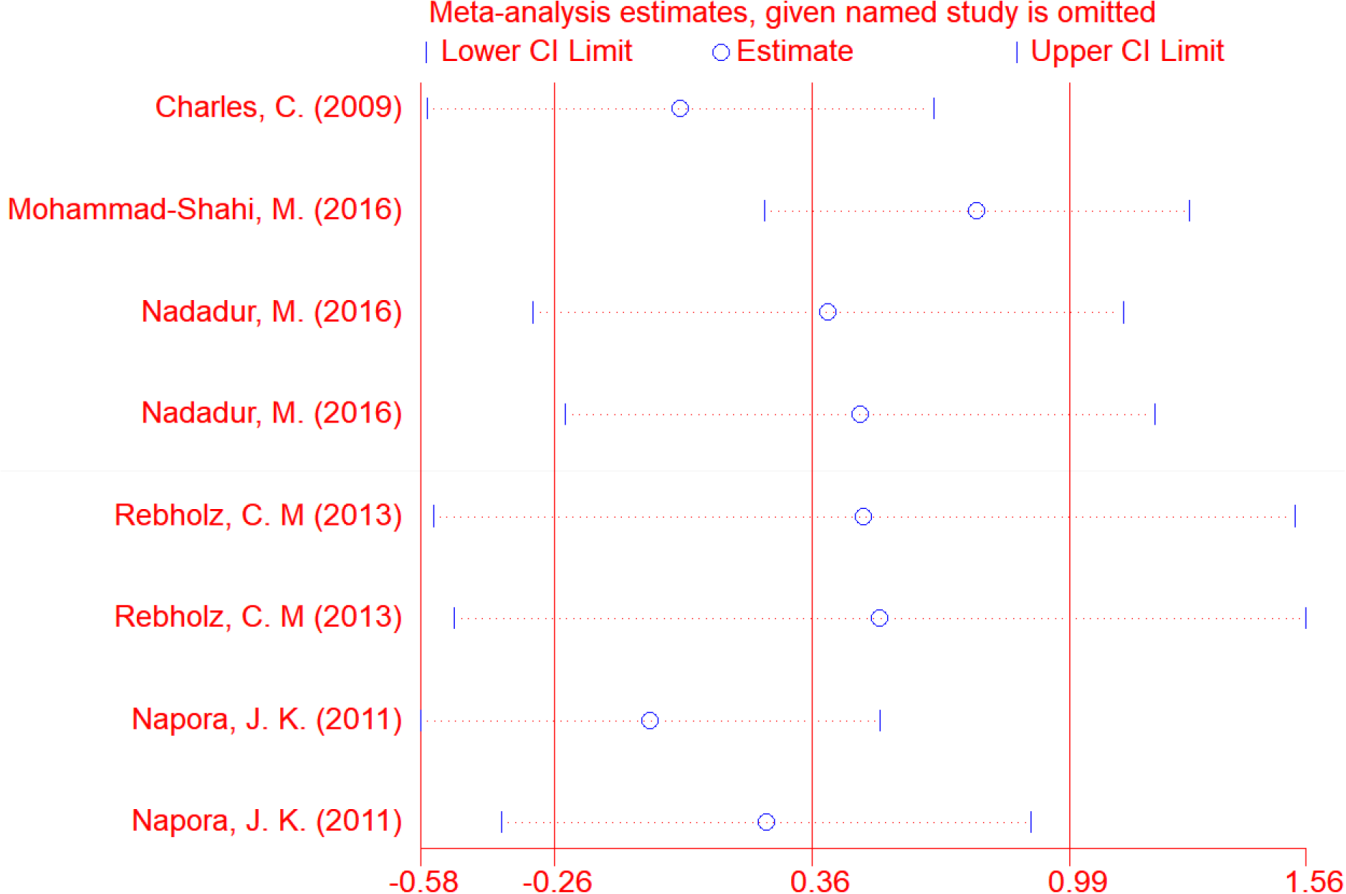
Sensitivity analysis for adiponectin

Eight effect sizes from five studies (Charles et al., 2009; Mohammad‐Shahi et al., 2016; Nadadur et al., 2016; Napora et al., 2011; Rebholz et al., 2013) were used to evaluate the overall effect of soy isoflavones combined to soy protein on serum adiponectin level (Figure 2). The overall estimate indicates that soy isoflavones in combination with soy protein had no significant effect on serum adiponectin level (weighted mean differences (WMD) = 0.36 μg/ml; 95% CI: −0.26, 0.99; *p* = .25). There was a significant heterogeneity among the included studies (Cochrane's Q test, *p* < .001; *I* ^2^ = 94.9%), and even it was found in most subgroup analyses except for study duration >56 days (*p* = .577; I^2^ = 0%), and baseline adiponectin ≤10 μg/ml (*p* = .107; *I* ^2^ = 50.7%) (Table 3). Depending on the results of sensitivity analysis, excluding no trials changed the overall effect of soy isoflavones in combination with soy protein on serum adiponectin level (Figure 3). Subgroup analysis revealed that soy isoflavones plus soy protein significantly increased serum adiponectin level in subgroups with soy isoflavones dose >90 mg/day (Mean change = 3.39, 95% CI: 1.20, 5.58; *p* = .002) (three effect sizes), soy protein dose >21 g/day (Mean change = 0.81, 95% CI: 0.14, 1.49; *p* = .018) (four effect sizes), studies with parallel design (Mean change = 1.84, 95% CI: 0.43, 3.25; *p* = .01) (five effect sizes), study duration >56 days (Mean change = 2.38, 95% CI: 1.55, 3.22; *p* ˂ .001) (two effect sizes), Americas (Mean change = 0.76, 95% CI: 0.25, 1.27; *p* = .003) (seven effect sizes), participants' age > 57 year (Mean change = 3.39, 95% CI: 1.2, 5.58; *p* = .002) (three effect sizes), BMI ≤29 (Mean change = 3.39, 95% CI: 1.2, 5.58; *p* = .002) (three effect sizes), and good quality studies (Mean change = 1.3, 95% CI: 0.59, 2.02; *p* ˂ .001) (five effect sizes) (Table 3).

**TABLE 3:** Subgroup analyses for studies evaluating the effect of soy isoflavones plus soy protein on serum adiponectin

	Subgroup	No. of trial	Change in adiponectin (95% CI)	*p*‐value	*I* ^2^ (%)	P_heterogeneity_
Total	–	8	0.36 (−0.26, 0.99)	.25	94.9	˂0.001
Soy isoflavones dose	≤90 mg/d	5	−0.46 (−0.98, 0.06)	.086	93.6	˂0.001
	>90 mg/d	3	3.39 (1.20, 5.58)	.002	84	0.002
Soy protein dose	≤21 g/d	4	−0.72 (−2.51, 1.06)	.428	96.5	˂0.001
	>21 g/d	4	0.81 (0.14, 1.49)	.018	94	˂0.001
Design	Parallel	5	1.84 (0.43, 3.25)	.01	93.9	˂0.001
	Cross‐over	3	−0.94 (−1.72, −0.16)	.019	96.7	˂0.001
intervention duration	≤56 days	6	−0.09 (−0.72, 0.54)	.785	95.2	˂0.001
	>56 days	2	2.38 (1.55, 3.22)	˂.001	0	0.577
Baseline adiponectin	≤10 μg/ml	4	0.01 (−0.17, 0.18)	.918	50.7	0.107
	>10 μg/ml	4	1.05 (−3.56, 5.66)	.655	97.4	˂0.001
Health status	Healthy	3	0.75 (−043, 1.94)	.214	92.5	˂0.001
	At risk/disease	5	0.1 (−0.75, 0.96)	.815	96.3	˂0.001
Sample size	≤44	4	1.62 (0.14, 3.11)	.032	93.4	˂0.001
	>44	4	−0.3 (−1.1, 0.5)	.466	96.6	˂0.001
Region	Americas	7	0.76 (0.25, 1.27)	.003	92.3	˂0.001
	Asia	1	−6.08 (−7.66, −4.5)	˂.001	–	–
Sex	Female	4	−0.72 (−2.51, 1.06)	.428	96.5	˂0.001
	Male	2	3.91 (−0.01, 7.83)	.05	88.9	0.003
	Both	2	0.01 (−0.22, 0.24)	.919	77.4	0.036
Age	≤57 years	5	−0.46 (−0.98, 0.06)	.086	84	0.002
	>57 years	3	3.39 (1.2, 5.58)	.002	84	0.002
BMI	≤29	3	3.39 (1.2, 5.58)	.002	84	0.002
	>29	3	−0.94 (−1.72, −0.16)	.019	96.7	˂0.001
	Unknown	2	0.00 (−0.41, 0.41)	.999	40.0	0.097
Quality assessment	Good	5	1.3 (0.59, 2.02)	˂.001	94.8	˂0.001
	Weak	3	−1.76 (−3.65, 0.13)	.068	96.5	˂0.001
Publication year of article	≤2010	1	2.5 (1.57, 3.34)	˂.001	–	–
	>2010	7	0.05 (−0.56, 0.66)	.875	94.5	˂0.001

Abbreviations: BMI, body Mass index; CI, confidence interval; mg/d, milligram per day; mg, milligram; μg/ml, microgram per milliliter.

**FIGURE 4:**
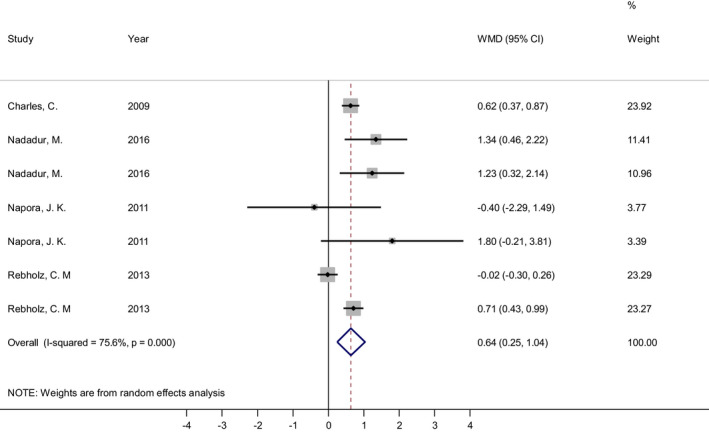
Forest plot of the effect of soy isoflavones plus soy protein consumption on serum concentrations of resistin

The effect of soy isoflavones combined to soy protein on serum resistin levels was evaluated using seven effect sizes from four studies (Charles et al., 2009; Nadadur et al., 2016; Napora et al., 2011; Rebholz et al., 2013) (Figure 4). The overall estimate indicated that soy isoflavones in combination with soy protein significantly increased serum resistin level compared with comparison group (WMD = 0.64 ng/ml, 95% CI: 0.25, 1.04; *p* = .001). There was a significant heterogeneity among the studies (Cochrane's Q test, *p* < .001; *I* ^2^ = 75.6%). In subgroup analysis, the heterogeneity among the studies was significant in the following subgroups: soy isoflavones dose ≤89.3 mg/day (*p* < .001; *I* ^2^ = 85.5%), soy protein dose >22 g/day (*p* < .001; *I* ^2^ = 92.3%), studies with cross‐over design (*p* < .001; *I* ^2^ = 92.3%), study duration≤56 days (*p* < .001; *I* ^2^ = 81.3%), baseline resistin ≤10.92 ng/ml (*p* < .001; *I* ^2^ = 81.9%), at risk individuals (*p* < .001; *I* ^2^ = 80.7%), sample size >37 (*p* < .001; *I* ^2^ = 87.4%), Americas (*p* < .001; *I* ^2^ = 75.6%), both sexes (*p* < .001; *I* ^2^ = 92.3%), age ≤ 57 years old (*p* < .001; *I* ^2^ = 85.5%), BMI > 29 (*p* < .001; *I* ^2^ = 92.3%), studies with good quality (*p* = .001; *I* ^2^ = 78.2%) and Publication year >2010 (*p* < .001; *I* ^2^ = 78.4%) (Table 4).

**TABLE 4:** Subgroup analyses for studies evaluating the effect of soy isoflavones plus soy protein on serum resistin

	Subgroup	No. of trial	Change in resistin (95% CI)	*p*‐value	*I* ^2^ (%)	P_heterogeneity_
Total	**–**	7	0.64 (0.25, 1.04)	.001	75.6	˂0.001
Soy isoflavones dose	≤89.3 mg/d	4	0.70 (0.10, 0.131)	.022	85.5	˂0.001
	>89.3 mg/d	3	0.62 (−0.01, 1.26)	.054	18.5	0.293
Soy protein dose	≤22 g/d	5	0.88 (0.40, 1.36)	<.001	35.2	0.187
	>22 g/d	2	0.34 (−0.37, 1.06)	.345	92.3	<.001
Design	Parallel	5	0.88 (0.40, 1.36)	<.001	35.2	0.187
	Cross‐over	2	0.34 (−0.37, 1.06)	.345	92.3	˂0.001
intervention duration	≤56 days	5	0.62 (0.05, 1.19)	.033	81.3	˂0.001
	>56 days	2	0.77 (−0.004, 1.55)	.051	23.6	0.252
Baseline resistin	≤10.92 ng/ml	5	0.64 (0.23, 1.05)	.002	81.9	<0.001
	>10.92 ng/ml	2	0.67 (−1.48, 2.83)	.541	59.2	0.117
Health status	Healthy	3	0.90 (0.40, 1.41)	<.001	46.1	0.157
	At risk/disease	4	0.4 (−0.25, 1.04)	.227	80.7	˂0.001
Sample size	≤37	4	1.16 (0.56, 1.77)	<.001	6.30	0.362
	>37	3	0.44 (0.00, 0.88)	.05	87.4	˂0.001
Region	Americas	7	0.64 (0.25, 1.04)	.001	75.6	˂0.001
Sex	Female	3	0.90 (0.40, 1.41)	<.001	46.1	0.157
	Male	2	0.67 (−1.48, 2.83)	.541	59.2	0.117
	Both	2	0.34 (−0.37, 0.24)	.345	92.3	<0.001
Age	≤57 years	4	0.70 (0.10, 1.31)	.022	85.5	<0.001
	>57 years	3	0.62 (−0.21, 1.26)	.054	18.5	0.293
BMI	≤29	3	0.62 (−0.01, 1.26)	.054	18.5	0.293
	>29	2	0.34 (−0.37, 1.06)	.345	92.3	˂0.001
	Unknown	2	1.29 (0.65, 1.92)	<.001	0.00	0.865
Quality assessment	Good	5	0.46 (0.03, 0.88)	0.034	78.2	0.001
	Weak	2	1.29 (0.65, 1.92)	<.001	0.00	0.865
Publication year of article	≤2010	1	0.62 (0.37, 0.87)	˂.001	**–**	**–**
	>2010	6	0.70 (0.14, 1.26)	.015	78.4	˂0.001

Abbreviations: BMI, body Mass index; CI, confidence interval; mg, milligram; mg/d, milligram per day; μg/ml, microgram per milliliter.

**FIGURE 5:**
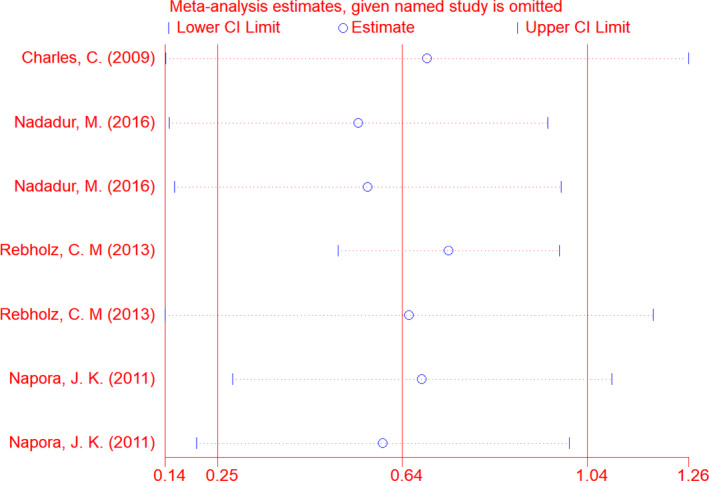
Sensitivity analysis for resistin

Concerning the sensitivity analysis results, excluding no trial, changed the overall effect of soy isoflavones in combination with soy protein on serum resistin levels (Figure **5**).The significant increasing effect of soy isoflavones in combination with resistin level was observed in the following subgroups: soy isoflavones dose ≤89.3 mg/day (*p* = .022), soy protein dose ≤22 g/day (*p* < .001), studies with parallel design (*p* <.001), study duration ≤56 days (*p* = .033), baseline resistin ≤10.92 ng/ml (*p* = .002), healthy individuals (*p* < .001), sample size ≤37 (*p* < .001), Americas (*p* = .001), females (*p* < .001), age ≤ 57 years old (*p* = 0.022), unknown BMI (*p* < .001), studies with good quality (*p* = .034), studies with weak quality (*p* < .001), publication year ≤2010 (*p* < .001), and publication year >2010 (*p* = .015) (Table 4).

**FIGURE 6:**
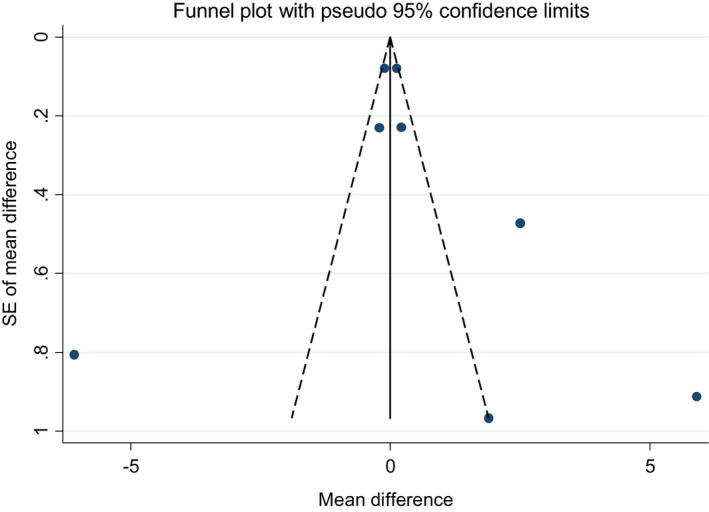
Funnel plots for the studies of the effects of soy isoflavones plus soy protein consumption on serum concentration of adiponectin

**FIGURE 7:**
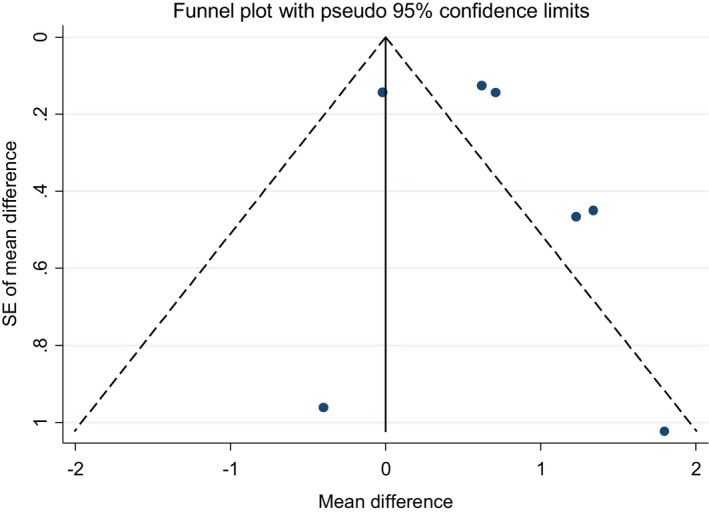
Funnel plots for the studies of the effects of soy isoflavones plus soy protein consumption on serum concentration of resistin

The funnel plots for studies on soy isoflavones in combination with soy protein on serum levels of both adiponectin and resistin were not visually symmetric (Figures 6 & 7). However, the results of Egger test and Begg test did not indicate any publication bias (Egger test *p*‐value = .606 and Begg test *p*‐value = .322 for adiponectin and Egger test *p*‐value = .466 and Begg test *p*‐value = .881 for resistin).

## 4 DISCUSSION

Our findings showed that soy isoflavones in combination with soy protein had no significant effect on adiponectin levels; however, studies with a parallel design, sample size ≤44, and good quality, studies on the American population, participants aged >57 years old, and BMI ≤29 showed significant incremental effects of soy isoflavones in combination with soy protein. A significant increase in adiponectin levels was observed in studies in which the dose of soy protein and soy isoflavones was more than the median (90 mg/day and 21 g/day, respectively).

Adiponectin was abundantly secreted and produced by adipose tissues and was widely recognized for its antiatherogenic, cardioprotective, antidiabetic, and anti‐inflammatory effects (Kern et al., 2003; Lee & Shao, 2014; Ohashi et al., 2012; Okamoto et al., 2002). The protective activity of adiponectin is shown in several inflammatory diseases, including cardiovascular diseases (CVDs), atherosclerosis, and insulin resistance (Daniele et al., 2011; Ouchi & Walsh, 2007; Villarreal‐Molina & Antuna‐Puente, 2012). Adiponectin gene expression and serum levels are both reduced with obesity, which might be the cause of inflammation (De Rosa et al., 2013). Anti‐inflammatory effects of adiponectin might be in terms of the stimulation of IL‐10 expression as an anti‐inflammatory cytokine, downregulation of inflammatory responses, and the suppression of nuclear factor kappa B (NF‐*κ*B) (Nigro et al., 2013; Ouchi et al., 2003; Ouchi & Walsh, 2007; Shibata et al., 2004).

Scientists proposed that soy supplementation inhibited the enhancement of total abdominal and subcutaneous fat and modified cardiometabolic risk factors (Akhlaghi et al., 2017; Mu et al., 2019; Zhubi‐Bakija et al., 2021). As a result, the decrease in adipose tissue after consuming soy products may result in an increase in adiponectin. The method via which soy reduced belly fat buildup is uncertain. The results of an animal study indicated that genistein, a soy isoflavone, in pharmacologic doses, inhibited the deposition of adipose tissue via the reduction of several factors responsible for adipose tissue differentiation (Penza et al., 2006). Regardless of the mechanism, our results indicated that soy isoflavones combined to soy protein nonsignificantly increased adiponectin levels.

We proposed three potential reasons why the nonsignificant results regarding the effect of soy isoflavones in combination of soy protein on adiponectin levels were obtained: soy isoflavones dose, study duration, and participants' BMI.

The results of our subgroup analysis revealed that soy isoflavones in combination with soy protein increased the serum levels of adiponectin in higher doses of isoflavones and protein. One animal study showed that soy isoflavones in pharmacological doses could reduce adipose tissue (Penza et al., 2006). Therefore, the anti‐obesity effect of soy isoflavones in high doses might enhance adiponectin. Another reason why the effect was significant might be in terms of higher serum concentrations of equol in high doses. Equol, as one of the soy isoflavones, is produced from daidzein and has antioxidant activity much higher than other isoflavones (Setchell & Clerici, 2010).

In trials with an intervention duration >56 days, the findings of our subgroup analysis demonstrated that soy isoflavones in conjunction with soy protein substantially enhanced the blood concentration of adiponectin. Out of eight trials, two trials had an intervention duration of more than 56 days; therefore, short intervention duration in most trials might be another reason for the nonsignificant effect of soy isoflavones in combination with soy protein on adiponectin levels.

Subgroup analysis revealed that soy isoflavones in combination with soy protein increased the serum adiponectin levels in studies which were conducted on subjects with BMI ≤29. It might be possible that the soy isoflavones in combination with soy protein cannot reduce adipose tissue in the obese population. The results of a meta‐analysis by Akhlaghi et al. (2017), indicated that soy products could reduce the waist circumference in overweight not obese subjects.

Subgroup analysis by area revealed that the combination of soy isoflavones and soy protein raised blood adiponectin concentrations in many American regions. The reason for this result might be a higher median intake of isoflavones in our study (90 mg/day) compared with normal Western diets in which mean intake is almost 2 mg/day (de Kleijn et al., 2001). However, further prospective RCTs in Asian and Western countries are needed to find the best dose of soy isoflavones to increase the adiponectin levels.

Resistin, a hormone secreted from adipose tissue, causes insulin resistance and leads to the development of T2DM (Kim et al., 2001; Steppan et al., 2001). Scientists have considered resistin to link obesity, especially visceral obesity, and diabetes (Tripathi et al., 2020). The association of resistin with metabolic disease is not limited to obesity or T2DM, it is associated with CVDs, hypertension, atherosclerosis, arthritis, and various malignancies (Filková et al., 2009). The results of our study revealed that soy isoflavones combined to soy protein increased the serum levels of resistin. In a few studies, it was found that the enhancement of resistin after taking soy products might be the cause of reported malignancy (Wang et al., 2021; Weng & Yuan, 2017). While interpreting our results, it should be kept in mind that results were from seven effect sizes with significant heterogeneity. Therefore, we could not report a firm conclusion.

Our study has limitations: First, there was a small number of qualified papers which were included in our study. Secondly, the duration of intervention was short. Only one study followed the subjects for more than 24 weeks. Thirdly, these articles reported no body composition changes (lean body mass, fat mass, and waist circumference) although adipokines were secreted from adipose tissue. Fourthly, there was only one article that studied the effect of soy isoflavones in combination with soy protein on adiponectin concentration in Asian participants and no article was on resistin levels among this population. Therefore, the effect of this combination on adiponectin and resistin levels in the Asian population remained unknown.

This research has many strengths: we conducted the search using a wide phrase. This investigation was conducted in accordance with the Cochrane Collaboration's principles for systematic reviews and meta‐analyses. We included studies that only used soy isoflavones combined to soy protein as an intervention. Therefore, we excluded the confounding effects. We have limited the effect of baseline adiponectin and resistin levels, soy isoflavones dose, design, soy protein dose, intervention duration, health status, sex, region, quality assessment, sample size, age, BMI, and publication year of the article on our results by subgroup analysis. We have conducted our systematic search with no time limitation.

## 5 CONCLUSION

The overall results of present review did not support the useful effect of soy isoflavones in combination with soy protein on serum adiponectin levels, but our study revealed that this combination could significantly increase the serum concentration of resistin. However, it appeared that this combination significantly increased the serum concentration of adiponectin in RCTs in which subjects took a higher dose of soy isoflavones and soy protein. Furthermore, high‐quality publications and articles among the American population indicated that this combination had a beneficial impact on the blood concentration of adiponectin. On the other hand, it seems that the dose of soy protein and soy isoflavones, RCTs quality, and the region of participants are strong predictors for this combination effect on serum adiponectin levels. In terms of the impact of this combination on resistin levels, further well‐designed intervention trials and studies conducted in diverse regions of the globe are needed to validate the rising effects of soy isoflavones in conjunction with soy protein on resistin levels.

## FUNDING INFORMATION

This study was financially supported by Neyshabur University of Medical Sciences.

## CONFLICT OF INTEREST

No conflict of interest.

## Supporting information


Table S1

## Data Availability

The data that support the findings of this study are available from the corresponding author upon reasonable request.
