# Supplementary material for: Can soy isoflavones in combination with soy protein change serum concentration of adiponectin and resistin? A systematic review and meta‐analysis on randomized clinical trials
Source: Food Sci Nutr. 2022 Sep 15;10(12):4126–38. doi: 10.1002/fsn3.3038 (PMC9731533; doi:10.1002/fsn3.3038)
Supplement: Supplementary file 1 — Table S1 [file FSN3-10-4126-s001.docx]

| **Supplementary table1.** | |
| --- | --- |
| Databases | Search Strategy |
| PubMed | (((**"Adipokines"[Mesh]** OR "Adipokines"[tiab] OR "Adipocytokines"[tiab] OR "Adipokine"[tiab] OR **"Adiponectin"[Mesh]** OR "Adiponectin"[tiab] OR "Adipocyte Complement-Related Protein 30-kDa"[tiab] OR "ACRP30 Protein"[tiab] OR **"Leptin"[Mesh]** OR "Leptin"[tiab] OR "Obese Protein"[tiab] OR "Obese Gene Product"[tiab] OR "Ob Gene Product"[tiab] OR "Ob Protein"[tiab] OR **"Ghrelin"[Mesh]** OR "Ghrelin"[tiab] OR "Obestatin"[tiab] OR "Appetite"[tiab] OR "Regulating Hormone"[tiab] OR "Motilin-Related Peptide"[tiab] OR **"Resistin"[Mesh]** OR "Resistin"[tiab] OR "Visfatin"[tiab] OR Adipok*[tiab] OR "Adipokines" OR "Adipocytokines" OR "Adipokine" OR "Adiponectin" OR "Adipocyte Complement-Related Protein 30-kDa" OR "ACRP30 Protein" OR "Leptin" OR "Obese Protein" OR "Obese Gene Product" OR "Ob Gene Product" OR "Ob Protein" OR "Ghrelin" OR "Obestatin" OR "Appetite" OR "Regulating Hormone" OR "Motilin-Related Peptide" OR "Resistin" OR "Visfatin" OR Adipok*) AND (**"Soy Foods"[Mesh]** OR "Soy Foods"[tiab] OR "Soy Food"[tiab] OR "Soy, Food"[tiab] OR "Soy, Foods"[tiab] OR "Soyfood"[tiab] OR "Soyfoods"[tiab] OR "Foods Soy"[tiab] OR "Soy Cheese"[tiab] OR "Soy Cheeses"[tiab] OR "Soy Sauce"[tiab] OR "Soysauce"[tiab] OR "Soy Bean Curd"[tiab] OR "Texturized Soy Protein"[tiab] OR "Texturized Soy Proteins"[tiab] OR "Texturized Vegetable Protein"[tiab] OR "Soya"[tiab] OR "Natto"[tiab] OR "Tempeh"[tiab] OR "Tofu"[tiab] OR "Miso"[tiab] OR **"Soy Milk"[Mesh]** OR "Soy Milk"[tiab] OR "Milk Soy"[tiab] OR "Milk, Soy"[tiab] OR "Soy Beverage"[tiab] OR "Soy Beverages"[tiab] OR "Soy, Beverage"[tiab] OR **"Soybeans"[Mesh]** OR "Soybeans"[tiab] OR "Soybean"[tiab] OR "Soy Bean"[tiab] OR "Soy Beans"[tiab] OR "Glycine max"[tiab] OR **"Soybean Proteins"[Mesh]** OR "Soybean Proteins"[tiab] OR "Soy Bean Proteins"[tiab] OR "Soy Protein"[tiab] OR "Soy Proteins"[tiab] OR "Proteins Soy"[tiab] OR "Protein Soy"[tiab] OR "Genistein"[Mesh] OR "Genistein"[tiab] OR "Soy Products"[tiab] OR **"Isoflavones"[Mesh]** OR "Isoflavones"[tiab] OR "Isoflavone"[tiab] OR "Homoisoflavones"[tiab] OR "3-Benzylchroman-4-Ones"[tiab] OR "3-Benzylidene-4-Chromanones"[tiab] OR **"Phytoestrogens"[Mesh]** OR "Phytoestrogens"[tiab] OR "Phytoestrogen"[tiab] OR "Phyto-Estrogen"[tiab] OR "Plant Estrogen"[tiab] OR "Plant Estrogens"[tiab] OR "Equol"[Mesh] OR "Equol"[tiab] OR Soy[tiab] OR "Soy Foods" OR "Soy Food" OR "Soy, Food" OR "Soy, Foods" OR "Soyfood" OR "Soyfoods" OR "Foods Soy" OR "Soy Cheese" OR "Soy Cheeses" OR "Soy Sauce" OR "Soysauce" OR "Soy Bean Curd" OR "Texturized Soy Protein" OR "Texturized Soy Proteins" OR "Texturized Vegetable Protein" OR "Soya" OR "Natto" OR "Tempeh" OR "Tofu" OR "Miso" OR "Soy Milk" OR "Milk Soy" OR "Milk, Soy" OR "Soy Beverage" OR "Soy Beverages" OR "Soy, Beverage" OR "Soybeans" OR "Soybean" OR "Soy Bean" OR "Soy Beans" OR "Glycine max" OR "Soybean Proteins" OR "Soy Bean Proteins" OR "Soy Protein" OR "Soy Proteins" OR "Proteins Soy" OR "Protein Soy" OR "Genistein" OR "Soy Products" OR "Isoflavones" OR "Isoflavone" OR "Homoisoflavone" OR "3-Benzylchroman-4-Ones" OR "3-Benzylidene-4-Chromanones" OR "Phytoestrogens" OR "Phytoestrogen" OR "Phyto-Estrogen" OR "Plant Estrogen" OR "Plant Estrogens" OR "Equol" OR Soy)) AND ("Clinical Trials as Topic"[Mesh] OR "Clinical Trial"[Publication Type] OR "RTC"[tiab] OR Random*[tiab] OR Trial*[tiab] OR Intervent*[tiab] OR "Cross-Over Studies"[Mesh] OR "Cross-Over Studies"[tiab])) |
| Scopus | TITLE-ABS-KEY ((("Adipokines" OR "Adipocytokines" OR "Adipokine" OR "Adiponectin" OR "Adipocyte Complement-Related Protein 30-kDa" OR "ACRP30 Protein" OR "Leptin" OR "Obese Protein" OR "Obese Gene Product" OR "Ob Gene Product" OR "Ob Protein" OR "Ghrelin" OR "Obestatin" OR "Appetite" OR "Regulating Hormone" OR "Motilin-Related Peptide" OR "Resistin" OR "Visfatin" OR Adipok*) AND ("Soy Foods" OR "Soy Food" OR "Soy, Food" OR "Soy, Foods" OR "Soyfood" OR "Soyfoods" OR "Foods Soy" OR "Soy Cheese" OR "Soy Cheeses" OR "Soy Sauce" OR "Soysauce" OR "Soy Bean Curd" OR "Texturized Soy Protein" OR "Texturized Soy Proteins" OR "Texturized Vegetable Protein" OR "Soya" OR "Natto" OR "Tempeh" OR "Tofu" OR "Miso" OR "Soy Milk" OR "Milk Soy" OR "Milk, Soy" OR "Soy Beverage" OR "Soy Beverages" OR "Soy, Beverage" OR "Soybeans" OR "Soybean" OR "Soy Bean" OR "Soy Beans" OR "Glycine max" OR "Soybean Proteins" OR "Soy Bean Proteins" OR "Soy Protein" OR "Soy Proteins" OR "Proteins Soy" OR "Protein Soy" OR "Genistein" OR "Soy Products" OR "Isoflavones" OR "Isoflavone" OR "Homoisoflavone" OR "3-Benzylchroman-4-Ones" OR "3-Benzylidene-4-Chromanones" OR "Phytoestrogens" OR "Phytoestrogen" OR "Phyto-Estrogen" OR "Plant Estrogen" OR "Plant Estrogens" OR "Equol" OR Soy*) AND ("Clinical Trials" OR "Clinical Trial" OR "RTC" OR Random* OR Trial* OR Intervent* OR "Cross-Over" OR "Cross Over" OR "Crossover"))) |
| Web of Sciences | TS= ((("Adipokines" OR "Adipocytokines" OR "Adipokine" OR "Adiponectin" OR "Adipocyte Complement-Related Protein 30-kDa" OR "ACRP30 Protein" OR "Leptin" OR "Obese Protein" OR "Obese Gene Product" OR "Ob Gene Product" OR "Ob Protein" OR "Ghrelin" OR "Obestatin" OR "Appetite" OR "Regulating Hormone" OR "Motilin-Related Peptide" OR "Resistin" OR "Visfatin" OR Adipok*) AND ("Soy Foods" OR "Soy Food" OR "Soy, Food" OR "Soy, Foods" OR "Soyfood" OR "Soyfoods" OR "Foods Soy" OR "Soy Cheese" OR "Soy Cheeses" OR "Soy Sauce" OR "Soysauce" OR "Soy Bean Curd" OR "Texturized Soy Protein" OR "Texturized Soy Proteins" OR "Texturized Vegetable Protein" OR "Soya" OR "Natto" OR "Tempeh" OR "Tofu" OR "Miso" OR "Soy Milk" OR "Milk Soy" OR "Milk, Soy" OR "Soy Beverage" OR "Soy Beverages" OR "Soy, Beverage" OR "Soybeans" OR "Soybean" OR "Soy Bean" OR "Soy Beans" OR "Glycine max" OR "Soybean Proteins" OR "Soy Bean Proteins" OR "Soy Protein" OR "Soy Proteins" OR "Proteins Soy" OR "Protein Soy" OR "Genistein" OR "Soy Products" OR "Isoflavones" OR "Isoflavone" OR "Homoisoflavone" OR "3-Benzylchroman-4-Ones" OR "3-Benzylidene-4-Chromanones" OR "Phytoestrogens" OR "Phytoestrogen" OR "Phyto-Estrogen" OR "Plant Estrogen" OR "Plant Estrogens" OR "Equol" OR Soy*) AND ("Clinical Trials" OR "Clinical Trial" OR "RTC" OR Random* OR Trial* OR Intervent* OR "Cross-Over" OR "Cross Over" OR "Crossover"))) |
